# Herbal medicine and diabetic kidney disease

**Published:** 2013-01-01

**Authors:** Saeed Mardani, Hamid Nasri, Mahmoud Rafieian-Kopaei, Shabnam Hajian

**Affiliations:** ^1^Department of Nephrology, Shahrekord University of Medical Sciences, Shahrekord, Iran; ^2^Department of Nephrology, Division of Nephropathology, Isfahan University of Medical Sciences, Isfahan, Iran; ^3^Medical Plants Research Center, Shahrekord University of Medical Sciences, Shahrekord, Iran

**Keywords:** Herbal medicine, Diabetic nephropathy, End-stage renal disease

**Implication for health policy/practice/research/medical education:**

Diabetic kidney disease is the leading cause of end-stage renal failure worldwide, however current treatments remain suboptimal. Recently various plants have shown beneficial effects not only on kidney function in diabetes mellitus, but also on kidney toxicities induced by some drugs or toxins. The active substances recognized in these plants include polysaccharides, flavonoids, xanthones and peptides.

Diabetic kidney disease is the leading cause of end-stage renal failure worldwide, however current treatments remain suboptimal. In fact, diabetes mellitus is a common chronic disease affecting many of people (1). Persistent hyperglycemia can damage the kidneys and leads to retinopathy through various mechanisms. Diabetic kidney disease is also linked to increased cardiovascular mortality. Clinical evidences suggest that there is no curative or preventive treatment for nephropathy of diabetes. Hence, there is a need to find out reliable treatments to slow the progression of diabetic complications (1,2). Recently various plants have shown beneficial effects not only on kidney function in diabetes mellitus (1,2), but also on kidney toxicities induced by some drugs or toxins (1–3). Herbal medicine seems to be a reasonable source for new drugs. Sustained hyperglycemia leads to amplified oxidative stress and activation of polyol pathway which may cause inflammation and kidney injury (2–4). Several plant extracts with hypoglycemic properties and kidney protective activities have been defined (4–6). It has been shown that metformin, a biguanide blood sugar regulatory compound from a herbal source (Galega officinalis), may be useful in the prevention of kidney injury (3–7). Some other herbal medicines such as curcumin from Curcuma longa, *Panax quinquefolium*, *Vitis vinifera* and glycosides from *Stelechocarpus cauliflorus* have also been shown to protect renal damage (2–5). Some herbal drugs have a positive influence on glucose homeostasis in diabetic patients. These plants have compounds effective on diabetes mellitus or impaired glucose tolerance. Some other plants lower blood pressure or improve the kidney and cardiovascular functions which are often disturbed in diabetic patients (6–8). The active substances recognized in these plants include polysaccharides, flavonoids, xanthones and peptides. There are various mechanisms by which reno-protection may be achieved, anti-oxidative properties seem to be very important. Renal injury is usually associated with an increase in oxidative stress and the oxidative stress induced renal damage is reduced by antioxidants. Increased activities and levels of antioxidants usually reduce in renal damage (4–9). Antioxidants usually give electrons to free radicals. People with low intake of vegetables and fruits have been shown to be at more risk for development of renal injury. Although free radicals contribute to renal injury, atherosclerosis and diabetes however, clinical trials do not uniquely confirm a substantial impact on renal injury (5–10). It seems that antioxidant in vegetables, fruits, and grains help preventing renal injury, also there is some proofs that taking single antioxidants such as vitamin E or vitamin C protect against kidney injury. The findings about combination antioxidants are also complicated and not entirely clear (5–11). Natural whole products, such as vegetables and fruits, seem to act as parts of elaborate networks and therefore, no single antioxidant can do the work of the whole ones (9–11).

## Authors’ contributions

All authors contributed to the paper equally.

## Conflict of interests

The authors declared no competing interests.

## Ethical considerations

Ethical issues (including plagiarism, misconduct, data fabrication, falsification, double publication or submission, redundancy) have been completely observed by the authors.

## Funding/Support

None declared.
